# Engaging underrepresented groups in community physical activity initiatives: a qualitative study of *parkrun* in the UK

**DOI:** 10.1186/s12889-024-18314-2

**Published:** 2024-03-14

**Authors:** Helen Quirk

**Affiliations:** Sheffield Centre for Health and Related Research (SCHARR), School of Medicine and Population Health, University of Sheffield, 30 Regent Street, S1 4DA Sheffield, UK

**Keywords:** Parkrun, Outreach, Underrepresented, Running, Walking, Volunteering

## Abstract

**Background:**

Underrepresented groups, including racial/ethnic minority groups and individuals with low socioeconomic status face complex barriers to engaging in community-based health initiatives. This research uses *parkrun*, an outdoor, mass-participation, weekly physical activity and volunteering initiative, to explore the engagement strategies (‘outreach activities’) that have been used to promote the inclusivity and diversity of *parkrun* events.

**Methods:**

Ten adult *parkrun* Ambassadors who fulfilled volunteer roles that involved promoting *parkrun* to underrepresented groups in the UK were interviewed. Interviews took place via telephone or video call in April-July 2021. Interview transcripts were analysed thematically.

**Results:**

Engagement strategies implemented by Ambassadors varied from opportunistic promotion within communities to strategic negotiations at higher decision-making levels. Approaches were characterised by a community-centred focus that ensured community networks and assets were utilised. Stories were considered valuable indicators of successful outreach. A common challenge to outreach for Ambassadors was limited personal and organisational capacity that impeded the widescale scope, reach and scalability of *parkrun’s* engagement attempts.

**Conclusions:**

*Parkrun* Ambassadors have used a wide range of outreach activities at different levels of influence. A number of challenges to doing sustainable and effective outreach have been highlighted that need to be addressed. Working with and alongside communities where community-based health initiatives events take place to understand how to address inclusivity issues could contribute to greater participation by underrepresented groups.

**Supplementary Information:**

The online version contains supplementary material available at 10.1186/s12889-024-18314-2.

## Introduction

Physical inactivity is a pressing and longstanding public health problem [1–4]. Certain groups within the population engage in particularly low levels of activity compared to others, making them even more at risk of poorer health and lower life expectancy [5, 6]. Groups that are disadvantaged or less well represented in relation to others include but are not limited to, racial/ethnic minorities, individuals with low socioeconomic status, and individuals with long-term health conditions. These groups often face unique and complex barriers to mass-participation physical activity initiatives and are often underrepresented. The UK government has called for approaches using inclusive strategies for these groups who tend to be less physically active [7, 8].

In its Global Action Plan on Physical Activity 2018–2030, the World Health Organization (WHO) identified the need for physical activity opportunities that use public spaces and engage whole communities [9], where ‘community’ may refer to a places (e.g., neighbourhoods), cultures, identities or groups of mutual interest (e.g., faith groups). Existing physical activity initiatives, especially those that target whole populations, risk not reaching all communities without targeted outreach efforts. For example, mass participation physical activity events have the potential to attract whole populations, but are often one-off events limited to those who are already active [10–13]. Understanding how to address the barriers to engagement among more diverse groups is essential in achieving health equity. This project explores this within the context of a weekly global community-based initiative called *parkrun*.

### Parkrun

*parkrun* (written with a lower-case ‘p’) is the largest organisation of its kind in the UK providing regular outdoor community-based physical activity to whole communities. *parkrun* delivers free, weekly, organised 5-kilometre events and 2-kilometre ‘junior *parkrun*’ events for adults and children above the age of 4 in public spaces across the world (23 countries). The focus of this research is the UK. In the UK, the 5 km events take place across over 700 locations every Saturday morning and there are over 300 junior *parkrun* events every Sunday morning. *parkrun*’s mission statement “To create a healthier and happier planet”, is accompanied by the pledge: “*Free, for everyone, forever*.” [14].

*parkrun* events are promoted as being inclusive to people from all backgrounds and abilities and research evidence would support its perceived inclusivity [15–19]. Research has shown that *parkrun* attracts groups with traditionally lower levels of physical activity, such as women, ‘overweight’ and older populations [20]. The social nature of *parkrun* events and the social transaction and interactions it offers is known to be important for initiation and continued attendance [21, 22], making it uniquely different to walking/running alone. However, research suggests that participants tend to be of white ethnicity [19, 23, 24] and of higher socioeconomic status [20]. Common barriers to *parkrun* reported by *parkrun* participants in the UK, Australia and Ireland are the start time being inconvenient, feeling too unfit, illness or injury, childcare obligations and lack of time [25]. Whilst *parkrun* is praised for its consistent mode of delivery (at the same time, in the same place each week), its format does limit the ability to address time barriers, but placing emphasis on the opportunity to take part as a family (with children), to walk and to volunteer could go towards countering some of the common barriers to participation [25].

Barriers to organised physical activity often relate to socioeconomic disadvantage [26]. Research investigating the equitability of *parkrun* has found that people living in more deprived neighbourhoods of England live closer to *parkrun* events than people living in less deprived areas, yet participation is consistently lower in those from more deprived areas [27]. Findings from the *parkrun* UK Health and Wellbeing Survey 2018 found that participants who self-reported being physically inactive at *parkrun* registration and lived in the most deprived neighbourhoods benefitted the most from *parkrun* on measures of physical activity, overall health and wellbeing [28]. *parkrun* has been recognised in the WHO’s Global Action Plan on Physical Activity 2018–2030 as a working example of “*regular mass-participation initiatives in public spaces, engaging whole communities, to provide free access to enjoyable and affordable, socially and culturally appropriate experiences of physical activity*” (WHO; page 66). Yet it remains to be known what has and can be done to engage ‘whole communities’ in ‘culturally appropriate’ ways– including those who face barriers to participation and are underrepresented. Much of the *parkrun* research to date has focused on existing *parkrun* participants [21], leaving gaps in knowledge about non-participants and how *parkrun* can reach new and diverse audiences. Given that people from underrepresented groups could have the most to gain from participating in *parkrun* and its free-to-access and regular nature makes it a viable public health initiative, targeted outreach is needed so that it engages whole populations.

### Parkrun’s outreach work

*parkrun* have a worldwide network of Ambassadors who carry out volunteer roles, including outreach, that support *parkrun* event teams and *parkrun* Head Quarters, all of whom have undergone a robust recruitment process. *parkrun* describe Ambassadors as ‘*guardians, advocates, protectors and champions of parkrun*’ [29]. The ‘Outreach Ambassador’ role involves promoting the ways that people can become involved in *parkrun*, with a specific aim to ‘*help find ways to increase participation amongst groups that are underrepresented at [our] events*’ [30]. Outreach Ambassadors were first introduced in the UK in 2016 to help the expansion of junior *parkrun* events in the UK, including young people in socio-economically disadvantaged areas [31]. Different types of Ambassadors may also consider outreach to be within their remit of volunteering responsibilities such as Event Ambassadors (who support existing and new *parkrun* event teams) and Regional Ambassadors (who support Event Ambassadors across a region).

*parkrun’s* approach to outreach has never been studied and it is not clear what approach has been taken to increase participation amongst underrepresented groups. The aim of this study was to understand how community-based initiatives like *parkrun* can reach underrepresented groups. This study explored the perspectives of UK-based *parkrun* Ambassadors to understand what Ambassadors have done to engage with underrepresented groups and their perspectives of what works, what does not work and why.

## Methods

### Recruitment

A purposive sample of *parkrun* Ambassadors was recruited. Participants were eligible if they were based in the UK and their volunteer role involved planning or implementing outreach activities. There were no explicit exclusion criteria. Every UK Outreach Ambassador in role at the time across different locations of the UK was invited as well as additional Ambassadors who were known to be doing outreach work in their locality (approximately 15 Ambassadors were invited to be interviewed). Ambassadors were recruited via two *parkrun* staff members who contacted potential participants via email or during meetings with an invitation to take part. Those who expressed an interest to take part were encouraged to read the information sheet and give informed consent electronically. The personal characteristics (e.g., age, gender, ethnicity, locality and socio-economic status) of the ten Ambassadors interviewed were not collected to protect confidentiality.

### Data collection

Data collection took place between April and July 2021 from ten one-to-one semi-structured interviews. All except one took place via telephone which allowed geographic spread across the UK and was practical for the resource available to the researcher. One interview was conducted by video call at the Ambassador’s request. Interviews were recorded via a digital audio recorder and telephone microphone pick-up. An interview guide was reviewed by *parkrun* staff before being used (see supplementary material for interview guide). The interview guide provided structure and consistency across interviews but allowed flexibility to enable the participant to raise issues and shape the content and flow of the conversation. The researcher took notes during the interviews to act as prompts and to ensure that certain points of interest were returned to for more detail. These notes were not used as data in the analysis.

### Data analysis

Audio recordings of the interviews were transcribed verbatim. Analysis followed a similar thematic method to that outlined by Ritchie et al. [32] which involved an initial data management phase and subsequent abstraction and interpretation phase.

### Data management phase

Familiarisation was aided by the researcher conducting the interviews and listening back to recordings. Familiarisation continued with the reading and re-reading of transcripts systematically (transcript by transcript); highlighting words, phrases and paragraphs that represented salient attributes relevant to the research aim (codes). The familiarisation stage involved the construction of an initial thematic framework which helped organise the data– this was guided by important organising concepts: what’s been done, how has it been done, what have been the challenges. The software NVivo (version 11) was used to facilitate the organisation and management of the data. This early stage of coding has been described as ‘indexing’ [33] with the salient attributes acting as signposting to interesting bits of the data, rather than representing a final argument. In the early stages, the researcher stayed close to the original data transcripts (reading and re-reading transcripts in turn), allowing emergent ideas and concepts to be identified. As analysis progressed, the researcher coded the data in a more interpretative way (a process Ritchie et al., call ‘developing categories’). This involved attaching labels to the salient parts of the data and thinking about how they relate to the rest of the data within and between transcripts (development of initial themes). Throughout this process, the thematic framework was reviewed and refined.

### Abstraction and interpretation phase

After the data management process the researcher took stock and read back through the codes and initial themes. In this phase, the researcher sought to understand what was happening within each initial theme– looking at both the language (what was actually said by interviewees) and assigning meaning to what was said. Data and codes within themes were combined and rearranged, relabelled and new data was incorporated from the transcripts as required in an iterative manner. In this phase the researcher was also looking to identify linkage between the codes and initial themes. For example, at the individual case level, the researcher looked for possible connections between an experience (e.g., whether their Ambassador role aligned with their occupation) and a way of doing outreach (e.g., utilising their existing networks). The researcher also made links to existing knowledge, evidence or theory in this phase.

Themes and sub-themes were refined (subdivided and merged), reviewed and further refined and finally written up as findings. The write-up involved weaving, together the data extracts (i.e., verbatim quotations) with analytical narrative. This was not always a sequential process, but more fluid, with the researcher moving backwards and forwards between the data management and interpretation phases.

The researcher kept an analytic log (notes and memos) throughout the analysis process to keep a record of developing analytic thinking.

### Research team and reflexivity

For this study, it is important to acknowledge the interrelationship between the researcher, the data collected, the research participants and *parkrun*, as the organisation being researched. The researcher was trained and experienced in qualitative methods. They were female, aged 32 and White British, with no personal experience of living in or working in socio-economically deprived communities. They were not previously known to the interviewees, except for one participant who they knew from previous *parkrun* research correspondence.

The researcher had personal and professional experience with *parkrun*– both as a *parkrun* participant and as a researcher. Their personal and professional *parkrun* experience helped with developing rapport and building trust with interviewees and in establishing a collaborative relationship with *parkrun* staff. Their familiarity and interest with *parkrun* meant that they had a genuine desire to uncover the interviewees’ perspectives to better understand what Ambassadors have done to engage with underrepresented groups. During data collection and analysis, the researcher made conscious efforts to be critically reflexive about their personal and professional experience with *parkrun* to avoid imposing personal perspectives on the study, but instead use this to connect the data with their own ongoing experiences within the research context.

Members of *parkrun* staff were involved in the conceptualisation of the project and in the recruitment of participants. During data collection, the researcher did not discuss the interviews or findings with members of *parkrun* staff. During data analysis, the researcher had discussions with one member of *parkrun* staff about the project to provide an update and reflect on the project’s progress. *parkrun* staff did not have any involvement in the analysis of the interview data. The write-up of initial findings was shared with *parkrun* in the form of a project report. *parkrun* staff were not involved in the writing of this manuscript.

## Results

Out of the ten Ambassadors interviewed, seven were Outreach Ambassadors and three had other *parkrun* Ambassador roles; two Event Ambassadors and one Regional Ambassador. Ambassadors were working across the UK and many were working with *parkrun* events situated in more deprived communities (but their location was not recorded or redacted from transcripts to protect confidentiality). There was a range of experience among the Ambassadors interviewed from less than two years in the role to five or more years of experience. Interview length ranged from 42 to 99 min and the mean interview duration was 64 min.

The thematic analysis generated four main themes and nine sub-themes organised into two main organising concepts: *What parkrun’s outreach work involves* and *how parkrun’s outreach work is done.* See Table 1 for an overview of themes and sub-themes.

**Table 1:** Overview of themes and sub-themes, organised into two main organising concepts

Main organising concept	Main theme	Sub-theme
**What** ***parkrun*** **’s outreach work involves**	Theme 1 What outreach activities have been done to date?	1a) Outreach is about having conversations about *parkrun*
		1b) Three levels of outreach: on the ground, organisational and higher decision-making level
	Theme 2 What does successful outreach look like?	2a) Indicators of success: statistics and stories
		2c) Monitoring and reporting of progress: an ad hoc approach
**How** ***parkrun*** **’s outreach work is done**	Theme 3 What approach to outreach is taken?	3a) Different ways of working: opportunistic and strategic
		3b) A community-centred approach: Utilise existing connections, utilise existing assets and understand what the community needs
	Theme 4 What challenges are experienced when doing outreach?	4a) Personal capacity challenges: other commitments and responsibilities
		4b) *parkrun* capacity challenges: scope and reach
		4c) Broader inequity and inequality challenges: outside of parkrun’s control

### What *parkrun’s* outreach work involves

The first organising concept describes what Ambassadors were doing to engage with underrepresented groups.

### Theme 1

What outreach activities have been done to date?

#### 1a) Outreach is about having conversations about parkrun

Ambassadors were working within their own region across various parts of the UK and the boundary of their outreach area ranged from hyper-local (e.g., the Ambassador lived within or worked closely with the community) to regional (i.e., working across a whole region) or national. Whether hyper-local or regional, there was consensus that outreach involved raising awareness through effective communication and signposting to *parkrun (*“*spreading the word*” (P02) because this would have a “*ripple effect*” (P01) through communities. Ambassadors demonstrated willingness and confidence to talk and reach out to new people and communities. Being a good communicator was said to also include being a “*good listener*” (P07) and sometimes a “*persuader*” (P08). Talking about *parkrun* ranged from informal conversations with members of the public to formal presentation-style forms of communication (see Table 2 for specific examples of outreach activities). Ambassadors felt it was their role to challenge people’s perceptions about what *parkrun* is and who it is for; addressing some of the barriers that underrepresented groups may perceive or experience:

**Table 2:** Level of engagement and overview of outreach activities undertaken by parkrun Ambassadors

Level of engagement	Outreach activity	Description
*‘On the ground’ level*	Talking to people in the local community	Involved talking about *parkrun* and signposting people from the local neighbourhood or community to the nearby *parkrun* event. Talking to people involved informal conversations with members of the public and more formal conversations as part of organised events. *Informal conversation* included talking to members of the public about *parkrun* (e.g., on the bus, park users, neighbours). This also included helping to register people with *parkrun* (and on occasions, printing off their *parkrun* barcode). *Formal conversation* included attending events as a *parkrun* representative and/or having a stand/stall at events such as health events in community centres, employee wellbeing fairs and conferences.
	Hosting theme days and takeover days at local *parkrun* events	Involved organising theme days at their local *parkrun* event such as a ‘pyjama day’. These events were believed to get the best engagement from people and families who would not ordinarily attend. *parkrun* ‘takeover events’ (where a local club or community group run a *parkrun* for the day– providing all the volunteers on the day and promoting what their group does to the *parkrun* community) were another useful way of engaging with new people from the local community.
	Event activation	Involved doing outreach work as part of *parkrun* event activation (the set up and start of new *parkrun* events). This could involve seeking new event locations or working alongside other *parkrun* volunteers to help in the setup of new events such as finding the right people from the local community to be on the event team.
	Buddy scheme	Involved recruiting children who were regular *parkrun* participants to volunteer to participate with a new child with the aim of making the new child’s first experiences of *parkrun* more positive.
*Organisational level*	General Practice (GP) Practices	Involved liaising with GP practices and encouraging them to sign up for the ‘*parkrun* practice’ initiative. Activities ranged from ‘having a chat’ with health practitioners, to putting flyers up in GP surgeries, to liaising with the Royal College of General Practitioners (see below re: engaging at a higher decision-making level).
	Schools	Involved promoting *parkrun*/junior *parkrun* via schools– such as doing interactive presentations about *parkrun* at school assemblies. It was suggested that outreach activities with school should find options that do not depend on parental engagement– for example, the buddy scheme mentioned above or working with youth groups who could provide transport and supervision for children to attend their local *parkrun*.
	Local organisations	Involved working with local organisations to engage with local communities including; foodbanks, friends of the park groups, housing associations, weight loss organisations, large local employers (such as travel companies), children’s groups (such as Cubs, Brownies and sports clubs), and hospital trusts.
*Higher decision-making level*	Local authorities	Involved establishing links with local authorities (e.g., the public health team or health and wellbeing services). Activities involving local authorities were mainly conversations with ‘good contacts’ and attending meetings which had varying success.
	School authorities	Involved working with the school or education authorities to align *parkrun*/junior *parkrun* with school health and wellbeing mandates. It was suggested that this could involve schools signing up as *parkrun* advocates (something akin to the *parkrun* practice initiative).
	Government	Involved talking to politicians and having establishing contacts in the government who were *parkrun* advocates.

*Some [barriers] which are quite hard to break down, for example in the name “parkrun”, so people believing that it’s not just a run, that you can walk and stuff like that. And, I think a lot of the myths around it and around running, and people’s abilities… the role for an* Outreach Ambassador *is to tell people that they’re not true, basically, sort of displaying what parkrun is really about, and that it is for everybody. (P07)*

It was believed that face-to-face conversation was required, and some Ambassadors felt that conversations needed to take place over numerous occasions to be effective (a “*drip drip drip*” effect– P04).

This approach came with the acceptance that many attempts at conversation would fail, but there was persistence among Ambassadors to keep trying. For example:

*Some things have gotta be done, like talking to housing associations and council departments. Some of it has bred good contacts, some of it has been a total, total waste of time. But if you don’t do it, you don’t know [laughs]… it might not be. And, you know, you might get somebody who can really swing something for us.* (P02)

#### 1b) three levels of outreach: on the ground, organisational and higher decision-making level

Ambassadors were having conversations about *parkrun* at different levels of influence. Table 2 provides examples of outreach activities initiated at each level. Some activities involved working purely ‘on the ground’ level, interacting with people in their local neighbourhood and communities. One Ambassador regarded *parkrun* event activation (the set up of new *parkrun* events) in areas of high deprivation as the single most effective outreach activity they had been involved in because this provided opportunity to find event team members who represent the local community.

Others described liaising with local organisations to reach communities. Common organisations that had been used to reach local communities were General Practices (GP) and Schools. Doing a presentation about junior *parkrun* in a school assembly had been a common outreach activity, but Ambassadors felt initial interest did not always translate into participation, for example: “*we’d get this lovely warm reception, but… there’d be no continuity afterwards.*” (P02). Ambassadors acknowledged that a more effective approach would be to engage directly with parents/guardians rather than children– because, “*if those parents don’t want to come along, the kids are not coming.”* (P01).

Some Ambassadors were working at a more strategic level– liaising with local, regional or national decision-makers such as local authorities and government representatives. One Ambassador felt *parkrun* should be working with education authorities to align *parkrun*/junior *parkrun* with school health and wellbeing mandates– which would see schools signposting teachers, children and their families to the local *parkrun* event (similar to the model adopted by the *parkrun* practice initiative [34]).

### Theme 2

What does successful outreach look like?

#### 2a) indicators of success: statistics and stories

Ambassadors shared their thoughts about what success looks like for *parkrun*’s outreach work. They described indicators of success as statistics and stories. In terms of statistics, Ambassadors wanted to see better representation of underrepresented groups at *parkrun* events (i.e., indicating greater diversity). They also wanted to see the number of *parkrun* events in deprived areas increase (i.e., growth). Ambassadors shared a concern that demonstrating the impact of outreach work was difficult because there was often no observable impact or benchmark upon which to measure success. The challenge of using statistics as indicators of success was that the markers of success were often unknown, for example:

*As a result of the outreach that we do, if we had double the average participation, this sounds like a 50% increase, yay! But we only had 12 kids to start with. And one time we had one [participant] and other times we’ve had two [participants]. So… we’re actually quite pleased with the numbers, for some it would be small but for us it’s like oh 22 kids that’s a good turnout. (P04)*

Whilst Ambassadors acknowledged the importance of statistics for *parkrun* for accountability with funders, they did not feel this pressure to meet targets or performance indicators, as explained:

*These big organisations that need to continue with funding, and in order to get funding you need to prove that, again, it’s working, that you’ve got the, you’ve got people engaged, you’ve got people coming to it. And, like, I think what’s nice in being a volunteer Outreach Ambassador [is] maybe having that step away from it and not having the pressure (P04)*.

Difficulty quantifying successful outreach meant there was a preference for using individual stories as markers of success. Ambassadors preferred to consider success by *“following the journey of an individual or group”* (P03). Stories were valued for being able to illustrate how people have overcome adversity or barriers to participation, how they have built relationships and any improvements to health experienced since participating in *parkrun*. The preference for stories over statistics is summarised in this comment: “*if we only had one participant but that one participant enjoys it [parkrun] and it makes a difference to their lives and they’ve got something out of it, then our job [as Ambassadors] is done.”* (P04).

#### 2b) monitoring and reporting of progress: an ad hoc approach

Ambassadors described an unstructured approach to monitoring and reporting of outreach activities. Some Ambassadors shared an online document to log activities whilst others preferred to keep a mental note or emails as an audit trail of useful contacts. Ambassadors described reporting back to *parkrun* via ad hoc conversations (e.g., when they had something pertinent to share) or via meetings with *parkrun* staff / Head Quarters (HQ). Meetings with *parkrun* HQ and other Ambassadors were said to provide good opportunities to report back and share learnings with others:

*There is wisdom to be gleaned isn’t there from people who, who have done it and who are doing it and, and I think there’s certainly principles and there’s things that have worked here I’m sure would translate into other places*. (P05)

Most Ambassadors were satisfied that the current unstructured way of monitoring and reporting progress worked well for the limited time they had available for their volunteer role, with some seeing the value in *parkrun* introducing something more structured like an information repository. There was a suggestion that more time could be prioritised by for “*reflection and learning to improve…looking in the rear-view mirror [rather than] default to go*” (P09).

### How *Parkrun’s* outreach work is done

The second organising concept captures how *parkrun*’s outreach programme is delivered from the perspective of Ambassadors.

### Theme 3

What approach to outreach is taken?

#### Theme 3a) different ways of working: opportunistic and strategic

Ambassadors described opportunistic and strategic ways of working and both were considered essential for *parkrun*’s outreach to work. Opportunistic approaches involved taking any opportunity to promote *parkrun* within local communities, such as, “*signing people up on the bus”* (P01) and talking to local park users about the event. One Ambassador described it as, “*I just chip away at my little corner of the bigger picture*” (P01), suggesting that Ambassadors often worked independently of the wider cohort of Ambassadors. Being willing to take opportunities to promote *parkrun* wherever they arise was deemed to be an important quality for an Ambassador.

Strategic approaches to outreach involved Ambassadors striving to align their outreach activities to *parkrun*’s current priority areas, or ‘the bigger picture’. For example:

*It has to be responsive to what’s going on and the organisation as well. What are the priorities, what are the big projects and how can the Outreach Ambassadors feed into that and how can you make the best use of the limited resource* (P04).

The strategic approach was a more considered effort, and one example involved an Ambassador who was working at a national level bringing together and overseeing a group of eleven passionate *parkrun* participants across the area who had the common goal of raising awareness of *parkrun* within communities that are underrepresented. This was considered a way of pooling limited resource, making sure there was a coherent effort of outreach activity across regions and enabled outreach work to have greater reach beyond small localities.

#### Theme 3b) a community-centred approach: utilise existing connections, utilise existing assets and understand what the community needs

Whether opportunistic or strategic, the approaches to outreach were centred on community. Many outreach activities were initiated because of existing contacts and connections Ambassadors held with stakeholders in the local community (i.e., connections they had before becoming a *parkrun* Ambassador). As one Ambassador said, “*it’s about getting to know people and using people– a horrible expression isn’t it, but nevertheless it’s true. You need to use your contacts*” (P02). Part of this approach was in utilising the existing *parkrun* community as an asset, as Ambassadors talked about links being made via people who were existing *parkrun* participants (e.g., existing *parkrun* participants who worked in the health or education sectors). However, Ambassadors spoke about the limitations of relying solely on existing connections, for example when contacts in certain fields or communities do not exist, or contacts are limited to a hyper-local area, rather than further afield. There is also a network continuity risk if just one Ambassador holds all the valuable connections and then retires from their role.

Ambassadors spoke about the importance of collaboration and working *with* communities. For example: *“working in collaboration, we can find the answers and share that insight and research to provide the best kind of support for people to enter parkrun”* (P03). They believed an important part of their role was to find people or organisations within communities who are influential, trusted and listened to by others and who could “*fly the flag*” for *parkrun* (P02, P06, P09). This was particularly important when Ambassadors were not embedded (lived or worked) within the communities being targeted. There was a sense that once these ‘champions’ or “pied pipers” (P09) had been identified, outreach became much easier because they were the gateway into the local community. As described:

*People who, are just good well-connected community people who, who not necessarily in terms of, you know, bringing in lots of people in, but are just able to connect other people maybe or who just have ability and social talent that enable a really good community to be built at your event. So, you know, just people that will talk to anyone and will make people feel welcome and will happily sort of signpost people to various other people or, do you know what I mean, just connecting people.* (P05)

The Ambassadors talked about the need to understand “*where the demand is*” (P01) within communities and to “*get under the skin of their thinking, their cultures, their religions, their lifestyle habits… to provide the best kind of support for people to enter parkrun*” (P03). Ambassadors wanted to fully understand the barriers to participation for underrepresented groups, referring to this as a “*bottom-up*” approach from within the community (P08). Ambassadors wanted the knowledge of community needs to be evidence-based, rather than speculative, but expressed concern that they (and *parkrun* as a whole) may lack capacity to fully understand what the need is, where the need is greatest and where to target resource; “*Well from an analytical point of view we don’t necessarily have good information about the landscape- where to direct our limited resources to. So basically the information is coming into us anecdotally*” (P09). Certain activities, such as helping people register with *parkrun* and printing off their unique participant barcode were said to address barriers for people whose first language was not English and who did not have access to the internet or a printer. Suggestions for future *parkrun* promotion campaigns included using TikTok and YouTube to appeal to younger audiences and working with faith groups to embed messages about *parkrun* from *within* communities. The time and resource needed to establish authentic connections with communities needs consideration. One Ambassador described how “*going out to a local mosque and going ‘why don’t you come along to parkrun?’ wouldn’t work*” (P08) because it takes time to build trust, rapport and establish relationships with communities.

The Ambassadors also identified a scalability challenge because “*no one size fits all*” (P04). Targeted, localised approaches made it difficult to “*lift and shift*” (P08) outreach activities that have worked in one context to another “*because everyone’s situation is so different*” (P04).

### Theme 4

What challenges are experienced when doing outreach?

#### Theme 4a) personal capacity challenges: other commitments and responsibilities

Personal challenges referred to the difficulties faced by Ambassadors due to conflicting commitments such as employment and family responsibilities. One Ambassador acknowledged that if they had more time, they would prioritise reflection and sharing their learning with other Ambassadors. Those who regarded themselves as having more time (e.g., because they were retired or worked part-time) felt able to commit more time to their outreach role, “*my main asset is I’m retired so I can do daytime meetings, which, a lot of Ambassadors can’t, and, you know, that must be a hindrance, I think*” (P02).

#### Theme 4b) parkrun capacity challenges: scope and reach

Some of the challenges discussed by Ambassadors related to *parkrun*’s organisational capacity. Many expressed a desire for more Ambassadors to be recruited to increase *parkrun*’s capacity for outreach work. This was considered important for the following reasons: (1) to have someone to work with (“*to bounce off their ideas*”; P01), (2) to make sure more regions were being covered, (3) to make sure community needs were being explored and addressed and (4) to ensure that opportunities for engaging with underrepresented groups were not being missed. Ambassadors often worked alone in their area or region, limiting the reach and scope of outreach that could be done. There was also a concern that not all areas of the country are covered by Ambassadors, limiting the collective impact. It was suggested that each *parkrun* event could have outreach volunteers on the event team to ensure adequate coverage across all *parkrun* events. Though Ambassadors acknowledged that for *parkrun HQ*, this would raise the challenge of managing a growing number of Ambassadors.

#### Theme 4c) broader inequity and inequality challenges: outside of parkrun’s control?

A final challenge for Ambassadors was the perception that *parkrun*’s outreach work was only a small part of a much larger challenge to tackle population health inequalities and that *parkrun* can only do so much to address wider inequity issues. For example, one Ambassador acknowledged that people living in deprived areas might not consider *parkrun* a priority; “*you’ve got children turning up to school with no shoes. So the fact that you’re trying to recruit them for a parkrun isn’t gonna happen because they haven’t got school shoes, never mind trainers*.” (P10). Another Ambassador had concerns about antisocial behaviours that might be more prevalent in deprived areas such as broken glass or syringes on the *parkrun* event route and the added risk or responsibility this gave to *parkrun* event teams.

Linked to this was the concern that the *parkrun’s* format might not be appropriate for everyone, especially groups who are currently underrepresented (for example, due to cultural barriers). Examples of where *parkrun* “*rules and regulations can be a little strict*” (P10) included the day and time of the events not being appropriate for certain groups, not having the resource to tailor or translate promotional materials to the local context and not being able to give a finish time to people who do not bring a *parkrun* barcode to be scanned when they have completed the event. Ambassadors suggested potential initiatives that might promote further inclusivity, for example, the *(not)parkrun* initiative (where people can submit a 5k walk or run on any route, day and time), a buddy scheme at junior *parkrun* events (“*where children who have been coming for a while would be a buddy for new child. So that child would have someone to run with if the parents didn’t want to run*” (P06)), events/initiatives for children that do not depend on parental involvement and working with organisations who might help with transport to *parkrun* events (e.g., Youth groups).

## Discussion

### Summary of findings

Mass-participation community-based health initiatives that are open-to-all face the challenge of engaging groups whose health would benefit the most. Targeted approaches to promotion and outreach are needed to reach underrepresented groups. This study has demonstrated that a range of outreach activities have been implemented by *parkrun* Ambassadors in the UK in attempt to increase engagement by more diverse groups. Activities ranged from ground-level community engagement to higher-level strategic engagement with decision-makers. A common characteristic of all outreach activities was raising awareness through conversations about *parkrun* with the ‘right people’ and engaging with community organisations. Ambassadors described opportunistic and strategic ways of working as important for remaining focused on a vision, but being flexible enough to respond to new opportunities whenever they arise. A common characteristic of all outreach attempts was finding key assets in the local community (community members, organisations, authorities) that can mobilise the community. Whilst the community engagement approach was valued, Ambassadors described not having the resource or capacity needed to understanding the needs of a local communities, establish authentic relationships and scale-up outreach activities.

Ambassadors described the difficulty of knowing whether their outreach activities were successful, but also acknowledged they lacked capacity to formally monitor and evaluate their activities. A monitoring, evaluation and learning framework that is built into the planning of outreach work and integrates qualitative and quantitative evidence, would allow Ambassadors to understand the impact of their efforts and leverage individual story-telling to portray the complexities of outreach but also learn from data being collected. Though striking a balance between stories and statistics often depends on the priorities of key stakeholders (Ambassadors, *parkrun*, funders), which needs to be explored further from the perspective of *parkrun* leadership team and funding bodies.

Ambassadors identified broader inequity challenges that made their outreach attempts more difficult, including lack of transport to *parkrun* events, which supports existing research that has found socio-political barriers to engaging underrepresented groups in organised, community-based physical activity for example, cost, travel, childcare and lack of time [19, 35, 36]. Furthermore, Ambassadors described a possible tension in striving for inclusivity when the format of events may not suit everyone.

### Implications of the findings in context of existing research

Previous research has demonstrated that for equitable participation in *parkrun* events in England, it is not enough to situate (more) *parkrun* events in areas of high socio-economic deprivation and expect this to translate into participation by people from the local neighbourhood [27] or underrepresented communities. A small number of *parkrun* studies have explored outreach and inclusivity issues [19, 37] and the current findings demonstrate the outreach activities that are possible. The outreach activities implemented by *parkrun* Ambassadors were consistent with practice in community development (utilising community assets and understanding the needs of local people) [38], but were limited in scope, reach and scalability by resource constraints. Raising awareness through word-of-mouth communication and working in partnership with community organisations who currently work with pre-identified groups (e.g., inactive people, people living in areas of high deprivation) have been found to be effective approaches to inclusive recruitment to community-based walking programmes in the UK [36, 39]. Word-of-mouth promotion can be effective for engaging with underrepresented groups, but needs to be tailored to the target networks (e.g., via partnerships with organisations such as schools, community organisations and faith groups) and not just to those who are socially well connected [39]. Utilising these productive partnerships and authentic relationships with communities can facilitate appropriate tailoring of outreach activities and build capacity, addressing the resource constraint issues often experienced by grassroots or voluntary-sector initiatives [39, 40].

As *parkrun* grows at scale across the world, sustainable and scalable approaches to outreach are needed and further research and evaluation is needed to understand whether *parkrun*’s outreach work and dependence on a volunteer workforce can achieve this. Outreach may involve developing supportive pathways into *parkrun* and a recent study has shown the potential value of the'Couch to 5 K' initiativeto attract previously inactive people into *parkrun* [41]. Further research is essential to understand other appropriate pathways into *parkrun* that meet the unique needs of different underrepresented groups. Exploring barriers to *parkrun* [25] would add to this evidence and should include qualitative methods to explore the perceptions of different underrepresented groups, including those not already registered with *parkrun*. This would help establish whether *parkrun* can diversify its current offer to be more accessible to more people, and whether changes to event format, structure or way of operating would be needed to adapt to local contexts and optimise inclusivity.

### Implications for policy and practice

The WHO has called for regular mass participation initiatives that engage whole communities with free, enjoyable, socially and culturally appropriate experiences of physical activity. Using *parkrun* as a real-world example allows for wider application of the findings to other community-based initiatives seeking more equitable participation, particularly those that rely on volunteers for delivery. The *parkrun* model exemplifies a health-promoting initiative that has the potential for scale and reach across whole communities. *Parkrun*’s open-to-all offer has meant that more targeted outreach is needed to reach those who would not ordinarily be exposed to existing promotional messages. Aspects of *parkrun’s* outreach work that were believed to improve engagement with underrepresented groups were consistent with practice in community development and a community-centred approach [42], where there is extensive evidence supporting its ability to improve population health and wellbeing and tackle health inequalities [43]. Findings suggest that organisations should aim to understand the needs of the target communities (bottom-up planning [44]), increase awareness about their initiative or programme in communities that are underrepresented, that this should be embedded from the very beginning (e.g., at event activation so that there is local ownership of the event), that local assets should be utilised to build trust in the community, and as part of this community connectors (residents or community members with strong social networks) should be identified to spread the word about the initiative and mobilise the community [45].

Collaborative work with people from the underrepresented groups is needed to develop genuine co-produced solutions to equitable participation. This takes time and resource and the commitment required may surpass the role of a volunteer. Outreach initiatives need to be evaluated so that organisations can determine what activities are needed, if/how they work (mechanisms of change), for who, why as well as how sustainable and scalable they are. The evaluation might challenge what type of evidence is considered appropriate, how evidence might be collected and whether alternative methods might be better able to make sense of complexity (e.g., participatory evaluation, theory of change, or realist evaluation) [46].

### Methodological considerations

The findings should be interpreted with consideration to the following methodological issues. The findings represent the views of self-selected Ambassadors and therefore people who are highly engaged with and supportive of *parkrun* and its outreach work. Given that the personal characteristics of Ambassadors (e.g., age, gender, ethnicity, locality, and socio-economic status) is likely to influence their outreach attempts and the challenges faced, collecting this information should be considered in future research. The research has not been able to uncover any important differences that may exist within or between different groups and the influence this has on the type of activities implemented and their success (e.g., outreach activities that would appeal to already active people from underrepresented groups may differ to activities that would engage inactive people from the same group). *parkrun’s* outreach work is likely to differ across different territories and so further research in other countries is needed to explore contextual and cultural differences. Future implementation and evaluation frameworks need to be able to highlight and account for this level of complexity.

## Conclusions

Community-based health initiatives wishing to reach and meaningfully engage with whole communities need to embed outreach and equity considerations into all aspects of their delivery model. *parkrun* Ambassadors have used a wide range of approaches to increase participation by people who are underrepresented at *parkrun* events. Several challenges to scalable and sustainable outreach have been highlighted such as personal and organisational capacity, broader inequity challenges and the need for an evaluation framework that can assess the success of outreach activities. Working with and alongside communities where community-based health initiatives take place is essential to design outreach approaches that meet the needs of the organisation and those being targeted.

### Electronic supplementary material

Below is the link to the electronic supplementary material.

Supplementary Material 1

## Data Availability

The data sets generated and analysed during the current study are not publicly available due to the sensitive and identifiable nature of our qualitative data, but further details are available from the corresponding author upon reasonable request.

## Declarations

### Author’s information

See the manuscript for discussion of information about the author that may aid the reader’s interpretation of the article, and understand the standpoint of the author.

## References

[1] Lee I-M, Shiroma EJ, Lobelo F, Puska P, Blair SN, Katzmarzyk PT (2012). Effect of physical inactivity on major non-communicable diseases worldwide: an analysis of burden of disease and life expectancy. Lancet.

[2] Guthold R, Stevens GA, Riley LM, Bull FC (2018). Worldwide trends in insufficient physical activity from 2001 to 2016: a pooled analysis of 358 population-based surveys with 1· 9 million participants. Lancet Global Health.

[3] Loyen A, Clarke-Cornwell AM, Anderssen SA, Hagströmer M, Sardinha LB, Sundquist K (2017). Sedentary time and physical activity surveillance through accelerometer pooling in four European countries. Sports Med.

[4] Sport England. Active Lives Survey 2021/2022 2022 [Available from: Active Lives Survey 2021/2022].

[5] Cavill N, Rutter H. The impact of interventions and policies on socio-economic status differentials in physical activity: Evidence review for the Health Equity Pilot Project (HEPP). 2017. https://ec.europa.eu/health/sites/health/files/social_determinants/docs/hepp_screport_physical_en.pdf

[6] Strain T, Sharp SJ, Spiers A, Price H, Williams C, Fraser C, et al. Population level physical activity before and during the first national COVID-19 lockdown: a nationally representative repeat cross-sectional study of 5 years of active lives data in England. Volume 12,100265. The Lancet Regional Health–Europe; 2022. 10.1016/j.lanepe.2021.100265 PMC8629728 34870255

[7] Department for Culture Media and Sport (2015). Sporting Future: a New Strategy for an active nation.

[8] Sport England. Uniting the movement: A 10-year vision to transform lives and communities through physical activity and sport [online][viewed 24 November 2021]2020.

[9] World Health Organization. Global Action Plan on Physical Activity 2018–2030: More Active People for a Healthier World: World Health Organization; 2018 [Available from: https://apps.who.int/iris/bitstream/handle/10665/272722/9789241514187-eng.pdf.

[10] Lane A, Murphy N, Bauman A (2013). An effort to ‘leverage’the effect of participation in a mass event on physical activity. Health Promot Int.

[11] Lane A, Murphy N, Smyth P, Bauman A (2010). Do Mass Participation Sporting events have a role in making populations more active? Research Report 2.

[12] Murphy N, Lane A, Bauman A (2015). Leveraging mass participation events for sustainable health legacy. Leisure Stud.

[13] McVinnie Z, Plateau CR, Lane A, Murphy N, Stevinson C (2023). Effects of engaging in mass participation sporting events on physical activity behaviour: a systematic review. Health Promot Int.

[14] parkrun Global Limited. parkrun Volunteer Hub: Mission Statement 2023 [Available from: https://volunteer.parkrun.com/principles/mission-statement.

[15] Hindley D. More than just a Run in the Park: An Exploration of Parkrun as a Shared Leisure Space. Leisure Sci. 2018:1–21.

[16] Stevinson C, Wiltshire G, Hickson M (2015). Facilitating participation in health-enhancing physical activity: a qualitative study of parkrun. Int J Behav Med.

[17] Morris P, Scott H. Not just a run in the park: a qualitative exploration of parkrun and mental health. Adv Mental Health. 2018:1–14.

[18] Grunseit A, Richards J, Merom D (2018). Running on a high: parkrun and personal well-being. BMC Public Health.

[19] Fullagar S, Petris S, Sargent J, Allen S, Akhtar M, Ozakinci G (2020). Action research with parkrun UK volunteer organizers to develop inclusive strategies. Health Promot Int.

[20] Stevinson C, Hickson M (2013). Exploring the public health potential of a mass community participation event. J Public Health.

[21] Grunseit AC, Richards J, Reece L, Bauman A, Merom D (2020). Evidence on the reach and impact of the social physical activity phenomenon parkrun: a scoping review. Prev Med Rep.

[22] Wiltshire G, Fullagar S, Stevinson C (2018). Exploring parkrun as a social context for collective health practices: running with and against the moral imperatives of health responsibilisation. Sociol Health Illn.

[23] Haake S, Quirk H, Bullas A. parkrun Health and Wellbeing Survey. 2018. https://awrcparkrunresearch.wordpress.com/2018/10/20/uk-ireland-parkrun-health-wellbeing-survey-2018-%e2%9c%93-output-available/: Advanced Wellbeing Research Centre, Sheffield Hallam University 2018.

[24] Smith R, Schneider P, Bullas A, Haake S, Quirk H, Cosulich R et al. Does ethnic density influence community participation in mass participation physical activity events? The case of parkrun in England. Wellcome Open Res. 2020;5. 10.12688/wellcomeopenres.15657.1 PMC7065574 32195360

[25] Reece L, Owen K, Graney M, Jackson C, Shields M, Turner G (2022). Barriers to initiating and maintaining participation in parkrun. BMC Public Health.

[26] Smith BJ, Thomas M, Batras D (2015). Overcoming disparities in organized physical activity: findings from Australian community strategies. Health Promot Int.

[27] Schneider PP, Smith RA, Bullas AM, Bayley T, Haake SS, Brennan A et al. Where should new parkrun events be located? Modelling the potential impact of 200 new events on socio-economic inequalities in access and participation. MedRxiv. 2019:19004143.

[28] Quirk H, Bullas A, Haake S, Goyder E, Graney M, Wellington C (2021). Exploring the benefits of participation in community-based running and walking events: a cross-sectional survey of parkrun participants. BMC Public Health.

[29] parkrun Global Limited. The Ambassador Programme 2023 [Available from: https://support.parkrun.com/hc/en-us/articles/360000567885-The-Ambassador-Programme.

[30] parkrun Global Limited. parkrun Volunteer Hub: Ambassadors 2023 [Available from: https://volunteer.parkrun.com/principles/ambassadors.

[31] parkrun Global Limited. Warburtons Boosts Junior parkrun 2023 [Available from: https://blog.parkrun.com/uk/2016/02/18/warburtons-boosts-junior-parkrun/.

[32] Ritchie J, Lewis J, Nicholls CM, Ormston R (2013). Qualitative research practice: a guide for social science students.

[33] Seale C (1999). Quality in qualitative research. Qualitative Inq.

[34] parkrun Global Limited. parkrun Volunteer Hub: parkrun Practice 2023 [Available from: https://volunteer.parkrun.com/principles/parkrun-practice-initiative.

[35] Withall J, Jago R, Fox KR (2011). Why some do but most don’t. Barriers and enablers to engaging low-income groups in physical activity programmes: a mixed methods study. BMC Public Health.

[36] Matthews A, Brennan G, Kelly P, McAdam C, Mutrie N, Foster C (2012). Don’t wait for them to come to you, you go to them. A qualitative study of recruitment approaches in community based walking programmes in the UK. BMC Public Health.

[37] Quirk H, Haake S (2021). Engaging people with long-term health conditions in a community-based physical activity initiative: a qualitative follow-up study evaluating the parkrun PROVE project. BMC Sports Sci Med Rehabilitation.

[38] Misener L, Schulenkorf N (2016). Rethinking the social value of sport events through an asset-based community development (ABCD) perspective. J Sport Manage.

[39] Hanson S, Cross J, Jones A (2016). Promoting physical activity interventions in communities with poor health and socio-economic profiles: a process evaluation of the implementation of a new walking group scheme. Soc Sci Med.

[40] Wildman JM, Valtorta N, Moffatt S, Hanratty B (2019). What works here doesn’t work there’: the significance of local context for a sustainable and replicable asset-based community intervention aimed at promoting social interaction in later life. Health Soc Care Commun.

[41] Relph N, Owen M, Moinuddin M, Noonan R, Dey P, Bullas A (2023). How can UK public health initiatives support each other to improve the maintenance of physical activity? Evidence from a cross-sectional survey of runners who move from couch-to-5k to parkrun. Health Promot Int.

[42] Public Health England. A guide to community-centred approaches for health and wellbeing. Public Health England London; 2015.

[43] Cassetti V, Powell K, Barnes A, Sanders T (2020). A systematic scoping review of asset-based approaches to promote health in communities: development of a framework. Global Health Promotion.

[44] Cleland CL, Hunter RF, Tully MA, Scott D, Kee F, Donnelly M (2014). Identifying solutions to increase participation in physical activity interventions within a socio-economically disadvantaged community: a qualitative study. Int J Behav Nutr Phys Activity.

[45] McKnight J, Kretzmann J. Building communities from the inside out. A path toward finding and mobilizing a community’s assets. 1993;9.

[46] Oatley C, Harris K (2021). How, and why, does participatory evaluation work for actors involved in the delivery of a sport-for-development initiative?. Managing Sport Leisure.

