# Supplementary material for: Engaging underrepresented groups in community physical activity initiatives: a qualitative study of parkrun in the UK
Source: BMC Public Health. 2024 Mar 14;24:804. doi: 10.1186/s12889-024-18314-2 (PMC10938745; doi:10.1186/s12889-024-18314-2)
Supplement: Supplementary file 1 — Supplementary Material 1 [file 12889_2024_18314_MOESM1_ESM.docx]

**Interviews with Outreach Ambassadors**

- Please describe your parkrun role
  - Purpose of the role?
- What motivated you to apply for this role?
- Can you describe your understanding of parkrun’s outreach work?
  - What is its overall aim/ambition?
  - Where do the team of Outreach Ambassadors fit in the bigger picture?
  - How is the outreach work being delivered / managed?

Looking back…

- How long have you been an Outreach Ambassador?
- What have you done as part of your role?
- Out of everything you’ve done, what has worked well/the best? Why?
  - What determines whether or not an activity has ‘worked’?
  - What prompted this activity?
  - What happened? / what impact did it have?
  - Who did you engage with? Why? How?
    - Prompt: What people/communities?
    - Prompt: Any organisations?
  - What resources were needed?
- What hasn’t worked well? Why?
- What have been the main challenges?
  - How have/could these be addressed?
- How do you know if what you’re doing is working?
  - In what way(s) do Outreach Ambassadors track or monitor the work they’re doing?
  - How are ideas and learnings shared? - among Ambassadors – to parkrun HQ?
- What qualities or skills are needed for you fulfil your role?
- What was your experience of the management, coordination and support you received from parkrun HQ?
  - Is there anything more that parkrun could have done to support them?
  - Would you change anything about how the outreach work is being delivered?

Looking forwards…

- What ambitions do you have for the future? What would you like to see happen?
- What would success look like to you?
- What support will you need to achieve this?
  - Is there anything more parkrun can do to support you?
  - Resources?
- What piece of advice would you give for other providers wanting to reach new audiences?

Other- COVID questions (if not already brought up)

- What opportunities / issues has COVID highlighted in terms of outreach?
- What role do Outreach Ambassadors have in the return of parkrun – in months/years to come?

Is there anything else you would like to say about your role as an Outreach Ambassador or about parkrun’s outreach work?
